# Did Chinese children with imaginary companions attribute more agencies to non-human items: Evidences from behavioral cues and appearance characteristics

**DOI:** 10.3389/fpsyg.2022.899047

**Published:** 2022-09-07

**Authors:** Lin Qiyi, Zhang Ruiyi, Zhang Yiwen, Zhou Nan

**Affiliations:** ^1^School of Education Science, Huaiyin Normal University, Huaian, China; ^2^School of Nursing and Delivering, Jiangsu College of Nursing, Huaian, China

**Keywords:** preschool children, pretend play, imaginary companion, agency attribution, behavioral cues, appearance characteristics

## Abstract

Previous studies have focused on the relationship between imaginary companions (ICs) and children’s social developments. As far as we know, few studies have focused on the relationship between ICs and children’s agency attributions. This study aimed to explore the potential differences in agency attributions between children with and without ICs, children with egalitarian IC relationships and hierarchical IC relationships. Children’s agency attributions were measured by two experiments. One was based on behavioral cues (Random animations/ToM animations) and the other was based on appearance characteristics (ball/doll). The results revealed that children with ICs attributed more cognitive properties to Random and ToM animations than children without ICs. Compared with children without ICs, children with ICs attributed marginally more biological properties to a ball and more psychological properties to a ball and a doll. However, children with egalitarian and hierarchical IC relationships did not differ in their agency attributions. The results suggest that children with ICs are more likely to attribute agencies to non-human items with behavioral cues or appearance characteristics than children without ICs. Compared with child-IC relationship qualities, IC status may be more related to children’s agency attributions. However, only a correlation between IC status and children’s agency attributions was found in this study and it is interesting for future researchers to investigate the potential causal directions between children’s IC status and their agency attributions. If one of the causal directions or both the causal directions exist, future researchers can further explore the underlying mechanism.

## Introduction

Attribution of agency, or the goal-directedness of agency, plays an important role in individual social development (Biro and Leslie, 2007). The ability to attribute mental states to other agents is the foundation of successful human interaction (Frith and Frith, 2010). Two factors influence agency attribution. They are behavioral cues and the agent’s appearance (Biro and Leslie, 2007). For example, compared with objects with rigid motion, preschool children attributed biological and psychological properties to objects with non-rigid caterpillar-like motion (Schlottmann et al., 2002). At present, experimental paradigm based on behavioral cues has been widely used to explore the differences between normal people and people with autism in verbal descriptions (Abell et al., 2000) and brain activation of the mentalizing systems (Castelli et al., 2000, 2002; Kana et al., 2009), which has triggered many new research hotspots.

As one important form of pretend play (Lin et al., 2018b), imaginary companion (IC) typically includes two types: personified object and invisible friend (Singer and Singer, 1990; Taylor, 1999; Gleason, 2017). Personified objects (POs) are objects that children animate and humanize (e.g., an animated and humanized Peppa Pig toy; Gleason et al., 2000). Invisible friends (IFs) are characters that do not actually exist, although the children treat them as though they do (Bouldin, 2006). Relationship between ICs and children’s social developments has been one of research mainlines around ICs (Lin et al., 2016). Some researchers focused on the differences between children with and without ICs in their agency attributions. As to behavioral cues, children with ICs were more likely to attribute biological properties to a triangle with random motion than children without ICs (Moriguchi et al., 2016b). Meyer and Tuber (1989) found that children with ICs had much more human, animal and inanimate movement responses than children without ICs. As to appearance characteristics, children with ICs attributed marginally more perceptual properties to a puppet and a human (both agents have faces) compared with children without ICs (Moriguchi et al., 2016b). Evidences also come from other cues. For instance, children with ICs were more likely to choose a puppet that made anthropomorphic statements (i.e., attributing psychological properties to non-human items) than a puppet who disagreed with the statements compared with children without ICs (Tahiroglu, 2012). In addition, some researchers focused on the influences of children’s agency attributions on their IC status. Moriguchi et al. (2016a) found that infants’ ability to predict non-human objects’ goal-directed actions predicted their IC status during preschool years. This result suggests that infants who tended to attribute psychological properties to non-human items may be more likely to create an IC compared with those who did not. The above results seem to support the notion that children with ICs may be more likely to attribute agencies to humans or non-human items than children without ICs.

However, this difference has been not found in some researches. For example, after watching animation movies that included interactions of geometric figures, children with and without ICs did not differ in their narratives when age and language ability were controlled (Tahiroglu, 2012). Moriguchi and Shinohara (2012) found that, children with ICs did not attribute more biological and perceptual properties to a human and a ball compared with children without ICs.

As to the differences between children with and without ICs in agency attributions, previous researchers proposed two explanations from the perspective of ICs. The first possible explanation is that children with ICs have a strong tendency to anthropomorphize compared with children without ICs (Tahiroglu, 2012). Specifically, IC play may have some positive effects on children’s simulations skills. During the interaction with ICs children may need to simulate some agency properties (i.e., how ICs think and feel) of POs or IFs in a given situation. The simulation skills may facilitate mentalizing (Harris, 2000). Thus, children with ICs can extend this anthropomorphic trend to other human (Roby and Kidd, 2008; Giménez-Dasí et al., 2014) and non-human agents (Moriguchi et al., 2016a).

The second possible explanation is that children with ICs have a more sensitive agent perception system than children without ICs (Moriguchi and Shinohara, 2012). Previous researchers argued that humans have an agent detection system which responds to real agents or non-human agents. These non-human agents have human-like or animal-like characteristics (Barrett, 2000; Boyer, 2002). This system may be sometimes overactive and responds to an agent with unusual characteristics (e.g., invisibility) (Guthrie, 1995; Boyer, 2002) or geometric figures (Heider and Simmel, 1944). As to ICs, the agent perception system is related to the creations of ICs or the interactions with ICs (Moriguchi et al., 2016b). Compared with the agent perception system of children without ICs, the agent perception system of children with ICs may respond to both real humans and imaginary agents (Moriguchi and Shinohara, 2012).

In general, we hold the opinions that having an IC, the tendency to anthropomorphize and sensitivity of the agent perception system may share the same core content-dual representation that is thinking about a representation in two different ways at the same time (Carlson and White, 2013). Specifically, dual representation is a central theme in contemporary theories of pretense (Carlson and White, 2013). Leslie (1987) proposed that when children pretend, they are making the distinction between what an object really is and its pretend identity. Since IC play is an instance of pretend play (Leslie, 1987), it may involve a lot of distinctions between reality and fantasy and children with ICs may have a good ability of dual representation. Meanwhile, when children have a stronger tendency to anthropomorphize or a more sensitive agent perception system, they may attribute more agencies (fantasy) to non-human items (reality), which also means they may have a good ability of dual representation. Thus, we speculate that children who have ICs may be more likely to have a stronger tendency to anthropomorphize or a more sensitive agent perception system compared with other children. However, it still has two possible situations. From one side, having an IC may have some positive effects on children’s tendencies to anthropomorphize or sensitivities of agent perception systems. From the other side, children who have a stronger tendency to anthropomorphize or a more sensitive agent perception system may be more likely to have an IC compared with other children.

Meanwhile, previous researchers proposed that one essential component of executive function (EF) is inhibition (Carlson and White, 2013) and inhibitory control is defined as the capacity to inhibit actions or thought processes that are not relevant to the task or goal at hand (Rothbart and Posner, 1985). Since we hold the opinions that having an IC, the tendency to anthropomorphize and sensitivity of the agent perception system may share the same core content-dual representation, they may have the same processes of inhibiting the reality of an object and giving some imagination to it at the same time. Thus, we speculated that having an IC, a stronger tendency to anthropomorphize and a more sensitive agent perception system may be beneficial to children’s developments of inhibitory control, which may result in better EF.

In terms of the relationship between children’s ICs and their social developments, previous researchers not only investigated the differences between children with and without ICs, but also explored the differences between different levels of ICs’ internal variables (IC types, child-IC relationship qualities) (Lin et al., 2018b). Two qualities usually exist in the relationships between children and their ICs: egalitarian relationships or hierarchical relationships (e.g., Gleason, 2004, 2017). Egalitarian IC relationships are similar to friendships (Gleason et al., 2000; Gleason, 2002). Children and their ICs are equal in status, and respect each other (Lin et al., 2018b). In the hierarchical IC relationships, children play the role of parents or the partners with more competence. Thus, ICs usually receive a great deal of guidance and nurturance from children (Gleason et al., 2000; Gleason, 2002). Children dominate these relationships (Lin et al., 2018b).

Previous researchers proposed that different child-IC relationship qualities may result in some differences in children’s social developments. Children with egalitarian IC relationships may have some advantages in social developments than children with hierarchical IC relationships (Gleason, 2004; Gleason and White, 2005). Previous studies found that children with egalitarian IC relationships had better peer relationships (Lin et al., 2018a) and social competence (Gleason and Kalpidou, 2014; Lin et al., 2018a), scored higher on the second-order false belief task (Lin et al., 2020b), and chose more social and constructive coping strategies (Gleason and Kalpidou, 2014) compared with children with hierarchical IC relationships. These results supported previous researchers’ notions.

However, to our knowledge, no studies have investigated the potential differences between children with egalitarian and hierarchical IC relationships in their agency attributions. We speculated that there may be differences in agency attributions between the two. Previous study found that IC types were not associated with child-IC relationship qualities and children with egalitarian IC relationships scored higher on the second-order false belief task than children with hierarchical IC relationships (Lin et al., 2020b). Given that more reciprocities exist in egalitarian IC relationships than in hierarchical IC relationships (Gleason and Kalpidou, 2014), previous researchers explained the difference that egalitarian IC relationships may have a higher demand for knowing mental states of ICs than hierarchical IC relationships (Lin et al., 2020b). Based on this idea, we speculated that children with egalitarian IC relationships may have a stronger tendency to anthropomorphize than children with hierarchical IC relationships. Children with egalitarian IC relationships may extend this anthropomorphic tendency to other agents. These processes may result in some differences in agency attributions between children with egalitarian and hierarchical IC relationships. In general, the work of investigating the potential differences in agency attributions between the two may help us have a better understanding of the relationship between the interactive processes of pretend play and children’s developments of social cognitions.

## The present study

The main purpose of this study was to further explore the relationship between children’s ICs and their agency attributions to non-human items. Two experiments were used to access children’s agency attributions. One was based on the behavioral cues and the other was based on the appearance characteristics.

Since the results of studies concerning the differences between children with and without ICs in agency attributions were somewhat inconsistent and none of these studies were from the Chinese cultural background, our first goal was to investigate the potential differences between children with and without ICs in their agency attributions to non-human items under a Chinese cultural background. Here, we adopted the procedure of Moriguchi and Shinohara (2012) and Moriguchi et al. (2016b). Based on previous researchers’ theory that children with ICs have a strong tendency to anthropomorphize than children without ICs (Tahiroglu, 2012), we predicted that children with ICs might attribute more biological, emotional and cognitive properties to non-human items with behavioral cues, and more biological, psychological and perceptual properties to non-human items with appearance characteristics than children without ICs.

Our second goal was to explore the potential differences between children with egalitarian and hierarchical IC relationships in their agency attributions to non-human items. Given that the reasons we had mentioned above, we predicted that children with egalitarian IC relationships might attribute more biological, emotional and cognitive properties to non-human items with behavioral cues, and more biological, psychological and perceptual properties to non-human items with appearance characteristics compared with children with hierarchical IC relationships.

## Materials and methods

### Participants

We used a convenience sampling method to recruit 105 children from a kindergarten in Jiangsu, China. In this study, one girl did not complete the peabody picture vocabulary test (PPTV; Dunn, 1965) and we deleted the data from this girl. Finally, all participants came from three classes and were composed of 56 males (age *M* = 63.11 ± 4.280 months) and 48 females (age *M* = 62.79 ± 3.632 months). The 4-year-old group included 25 participants (age *M* = 57.52 ± 1.686 months) and the 5-year-old group included 79 participants (age *M* = 64.68 ± 2.744 months). 77 children were first born (74.0%) and 59 children did not have siblings (56.7%). Almost all mothers of children identified their ethnicities as Han Chinese. Only one mother identified her ethnicity as Hui Chinese. Mothers’ education levels ranged from not having completed high school to having completed a postgraduate degree. The percentage of mothers who continue their education after finishing high school was 68.3%.

### Measures

#### Imaginary companion interview

The IC interview was based on a double-interview process (the children themselves, plus their mothers) proposed by Taylor and Carlson (1997). We also adopted some researchers’ improved procedures (Li, 2009; Lin et al., 2018a). During interviews conducted individually with children, research firstly used three monochrome pictures to tell the children two stories about ICs. One was about a PO (a stuffed bear) (Li, 2009; Lin et al., 2018a) and the other was about an IF called “Honghong” (Li, 2009). Then, the researcher asked the children whether he or she had one or more imaginary friends like the stuffed bear or Honghong at present or in the past. If the child answered “yes,” the researcher would ask he or she 7 questions about the details of the IC (i.e., the gender of the companions, how old were the companions, etc.) (see Li, 2009, for more details).

In order to save time, researcher only invited the mothers whose children reported that they have ICs to have an interview individually. At first, the researcher introduced the definition of an IC from Li (2009, p. 22) to the mother: IC, which is imagined by child and has distinct personality, usually includes two forms. One is totally imagined by children. We normally see the children mumbling with some words and pushing themselves to the play with somebody that we cannot see. The other is a toy which is anthropomorphized by the children and treated as a good playmate. Researcher also gave some examples about ICs. Then, researcher asked the mother whether she realized that her child had one or more ICs at present or in the past. If the mother answered “yes,” she would be asked 8 questions about the details of the IC (i.e., the name of the companion, the type of the companion: PO or IF, the gender of the companion) (see Li, 2009, for more details). In addition, these mothers also answered 8 questions about the child-IC relationship quality. These questions came from Gleason and Kalpidou (2014) [i.e., whether the child cared for, taught, disciplined, guided, praised the IC, whether the child-IC relationship was egalitarian or hierarchical (hierarchical relationship got 1point), whether the child was in charge]. The aggregate score of these questions created a *relationship quality* variable, wherein higher scores meant a more hierarchical child-IC relationship (Gleason and Kalpidou, 2014).

Imaginary companion interviews were coded by two coders. Only if both children and their mothers acknowledged the presence of an IC, and the description about ICs were consistent, children were considered to have an IC. Disagreements between coders were resolved by further discussions and *Kappa* between coders was 0.73. Within the children who were considered to have ICs, the aggregate score of the child-IC relationship quality questions reported by their mothers ranged from 1 to 7 (*M* = 4.48, SD = 1.504). Because of the normal distribution of scores, we used the mean as the standard to classify each child-IC relationship quality as egalitarian or hierarchical.

#### Experiment based on behavioral cues

In this study, we used the Frith-Happé animations (Castelli et al., 2000, 2002). We also adopted some other researchers’ procedures (Jipson and Gelman, 2007; Moriguchi et al., 2016b).

The experiment contains three types of animations. We thought that within these animations, ToM animations included the highest level of behavioral cues and Random animations included the lowest level of behavioral cues. Thus, we used the Random and ToM animations in this study. Both animations are presented by videos. Each video contains two triangles: a small blue triangle and a big red triangle. The two triangles move on a framed white background for about 30–40 s. Specifically, ToM animations include 4 videos that two triangles coax, mock, seduce and surprise one another. Random animations included 4 videos that two triangles bounce off the walls like the movement of billard balls, merely drift around, bounce off the walls and drift around, and bounce off the walls like the movement of tennis balls.

In order to further simplify the research procedures, we conducted a pilot study to evaluate children’s responses to the 4 videos which were in the same animations (ToM or Random). The evaluation was based on Frith’s appropriateness criteria and we emailed Frith’s research team asking for the criteria. The 4 videos in the Random animations were viewed by 16 children (8 boys and 8 girls). The 4 videos in the ToM animations were viewed by other 16 children (8 boys and 8 girls). The results revealed that there was no significant difference in children’s responses to the video in the same animations, *F*_1_ (1,15) = 1.38, *p* > 0.10, $\eta_{p}^{2}=0.08$ (Random); *F*_2_ (1,15) = 0.24, *p* > 0.50, $\eta_{p}^{2}=0.02$ (ToM), respectively. The results suggested that the videos in the same animations were almost homogeneous and we could select one of them randomly in the research procedures.

Random animations or ToM animations were presented to each child in two times, respectively. Children took part in the experiment individually. By using a computer program, researchers presented one of the 4 videos in the same animations randomly to child in each time. This computer program was written by one of the authors. After that, child was asked 9 questions about the small blue triangle in a fixed order. Specifically, three questions were about attributions of biological properties (“Does the triangle eat?”, “Does the triangle move on its own?”, “Does the triangle grow?”). Three questions were about attributions of emotional properties (“Can the triangle feel happy?”, “Can the triangle feel angry?”, “Can the triangle feel sad?”). Three questions were about attributions of cognitive properties (“Can the triangle think?”, “Can the triangle lie?”, “Can the triangle know the other triangle?”). For each question, positive answer got 1point and negative answer or no answer got 0 point.

#### Experiment based on appearance characteristics

In this study, the research procedures were similar to Moriguchi and colleagues’ research procedures (Moriguchi and Shinohara, 2012; Moriguchi et al., 2016b) and we made some revisions about the experiment stimuli.

The experiment included two stimuli: a white ball and a doll. Specifically, the ball had no face and other human-like physical characteristics. Only the shape of the ball was similar to a human’s head. The doll had a face and other human-like characteristics (e.g., it had four limbs and clothes). This experiment did not use a human as a stimulus. That is because a human might include some interference variables (e.g., some behavioral cues).

The ball or the doll was used as a stimulus in two times, respectively. Children took part in the research procedures individually. Specifically, researchers firstly asked the child some common questions in each time (e.g., “What toys do you like?”, “What colors do you like?”, “Who is your good friend?”), which might help to relieve children’s uneasy feelings. Then, researchers showed the child the ball or the doll and said: “This is my friend. We often play games together. It is my good friend.” This procedure might help child soon accommodate herself or himself to the experiment conditions and researcher had no more interaction with the ball or the doll in the following procedures. After that, researchers asked the child to repeat the words that he or she had just heard. Finally, the child was asked 9 questions about the ball or the doll in a fixed order. Three questions were about attributions of biological properties (“Does it walk?”, “Does it eat?”, “Does it grow?”). Three questions were about attributions of psychological properties (“Can it feel happy?”, “Can it feel angry?”, “Can it think?”). Three questions were about attributions of perceptual properties (“Can it see things?”, “Can it hear things?”, “Can it smell things?”). Scoring was the same as the experiment which was based on behavioral cues.

#### Peabody picture vocabulary test

The peabody picture vocabulary test (PPTV; Dunn, 1965) was used to access children’s receptive vocabulary. The Chinese version of the test that we used in this study was revised and standardized by Xinhua Hospital. The hospital is affiliated to the School of Medicine, Shanghai Jiao Tong University. Children took part in the test individually. The test can be used among children aged 2–18 years and the aggregate score ranges from 0 to 120.

#### Procedure

All the research procedures were completed in two separate rooms of the kindergarten. This study included 3 researchers (2 males and 1 female). Specifically, one male researcher completed the IC interview. One male researcher and one female researcher completed the procedures of the experiment that was based on behavioral cues and the experiment that was based on appearance characteristics. The procedures of PPTV were completed by two male researchers. Mothers did not know our research goals and hypotheses.

Children’s research procedures were completed over 6 sessions and each session lasted for about 15 to 30 min. The IC interview was administrated to children during the first session. Children completed the procedures of Random animations during the second session and ToM animations during the third session. Children completed the procedures of the ball during the fourth session and the doll during the fifth session. The PPTV was administrated to children during the sixth session. In each class, the order of children was according to the order in the attendance book. Part of mothers received the IC interview in the afternoon and the interview lasted for about 20 to 40 min. In each class, the order of mothers was negotiated by teachers and mothers.

This study was approved by the Academic Ethics Committee of Huaiyin Normal University. All the mothers were informed and they agreed themselves and their children to participate in the research procedures. All the mothers also completed one family questionnaire before the research procedures.

#### Statistical analysis of data

We used SPSS25.0 software to statistically analyze the data that was obtained in this study. The statistical methods used are mainly descriptive statistics: *χ*^2^-test, *t*-test, *F*-test and simple effect-test.

## Results

### Descriptive statistics and preliminary analyses

Researcher interviewed 41 mothers. As mentioned above, one girl did not complete the PPVT. We also deleted the results of her mother’s interview. Among these mothers, 6 mothers’ descriptions about ICs were not consistent with their children’s descriptions about ICs. 10 mothers said that their children did not have ICs at present or in the past.^1^ One IC which was mentioned by both mother and child was classified as a transition object. Finally, two coders classified 23 of the 104 children as having ICs (22.1%, 10 boys and 13 girls). 17 children had POs (16.3%, 8 boys and 9 girls), 4 children had IFs (3.8%, 2 boys and 2 girls), and 2 girls had both IFs and POs (1.9%). For children with both types of IC, we followed previous researchers’ procedures (Gleason et al., 2000; Lin et al., 2018a, 2020a) and only used the data from IFs.

In terms of IC status, we categorized all children into one of the two groups for analysis: children with ICs (*N* = 23) and children without ICs (*N* = 81). ICs status was not associated with gender [*χ*^2^(1, *N* = 104) = 1.28, *p* > 0.10, *φ* = 0.11], birth order [*χ*^2^(1, *N* = 104) = 0.27, *p* > 0.50, *φ* = −0.05] and age group [*χ*^2^(1, *N* = 104) = 0.72, *p* > 0.10, *φ* = 0.08].

Based on the child-IC relationship quality variable, 11children were identified as having hierarchical IC relationships (10.6%, 3 boys and 8 girls), and 12 children were identified as having egalitarian IC relationships (11.5%, 7 boys and 5 girls). In terms of child-IC relationship quality, we categorized all children with ICs into one of the two groups for analysis: egalitarian relationship (*N* = 12) and hierarchical relationship (*N* = 11). Within the children with ICs, child-IC relationship quality was not associated with gender (Fisher’s exact tests, *p* > 0.10, *φ* = −0.31), birth order (Fisher’s exact tests, *p* > 0.10, *φ* = 0.29), and age group (Fisher’s exact tests, *p* > 0.50, *φ* = −0.21). Within children with ICs, child-IC relationship qualities were not associated with IC types (Fisher’s exact tests, *p* > 0.50, *φ* = −0.03).

In conclusion, the factors (sex, birth order and age group) were not included in the further analysis.

### Measures of agency attributions to ToM and random animations

Table 1 showed the average score and standard deviation of children with and without ICs in their agency attributions to ToM and Random animations. Children with ICs scored higher on the PPTV than children without ICs (*t* (102) = −3.15, *p* < 0.01, *Cohens’ d* = −0.62). Thus, we conducted group (IC/NIC) × animation (ToM/Random) ANOVAs, respectively, on scores for each property (biological, emotional and cognitive) and PPTV scores were used as a covariate. The power (1-β) for the group was 0.946 (the same below). For cognitive properties, there was a significant main effect of group. This result indicated that children with ICs were more likely to attribute cognitive properties to ToM and Random animations compared with children without ICs. More details could be seen in Table 2.

**Table 1:** Mean and standard deviation of agency attributions to ToM and random animations for imaginary companion status.

	With ICs (*N* = 23)		Without ICs (*N* = 81)	
	*M*	SD	*M*	SD
Random-biological	1.83	0.937	1.49	1.062
Random-emotional	1.65	1.191	1.47	1.073
Random-cognitive	1.65	1.071	1.15	0.923
ToM-biological	1.96	1.107	1.62	1.135
ToM-emotional	1.78	1.204	1.57	1.095
ToM-cognitive	1.83	0.984	1.31	1.020

**Table 2:** Comparisons between children with and without ICs in agency attributions to ToM and random animations.

	Main effect of group			Main effect of animation			Interaction		
	*F*	*p*	$\eta_{p}^{2}$	*F*	*p*	$\eta_{p}^{2}$	*F*	*p*	$\eta_{p}^{2}$
Biological	1.393	0.241	0.01	0.833	0.364	0.01	0.042	0.838	0.00
Emotional	0.935	0.336	0.01	0.083	0.774	0.00	0.000	0.992	0.00
Cognitive	4.413*	0.038	0.04	0.123	0.727	0.00	0.026	0.871	0.00

* *p* < 0.05: Annotation.

Table 3 showed the average score and standard deviation of children with egalitarian and hierarchical IC relationships in their agency attributions to ToM and Random animations. Children with egalitarian and hierarchical IC relationship did not differ on the PPTV scores (*t* (21) = −0.46, *p* > 0.50, *Cohens’ d* = −0.20). Thus, this variable was not included in the following analysis relating to child-IC relationship qualities. Relationship (egalitarian/hierarchical) × animation (ToM/Random) ANOVAs were conducted on scores for each property (biological, emotional and cognitive), respectively. The power (1-β) for the relationship was 0.366 (the same below). The power was relatively low and the results relating to the relationship should be interpreted with caution. For each property, the main effects of animation, relationship and the interaction between relationship and animation were not significant. More details could be seen in Table 4.

**Table 3:** Mean and standard deviation of agency attributions to ToM and random animations for child-IC relationship quality.

	Egalitarian relationship (*N* = 12)		Hierarchical relationship (*N* = 11)	
	*M*	SD	*M*	SD
Random-biological	1.67	0.985	2.00	0.894
Random-emotional	1.50	1.243	1.82	1.168
Random-cognitive	1.58	0.996	1.73	1.191
ToM-biological	1.58	1.084	2.36	1.027
ToM-emotional	1.58	1.311	2.00	1.095
ToM-cognitive	1.50	0.798	2.18	1.079

**Table 4:** Comparisons between children with hierarchical and egalitarian IC relationships in agency attributions to ToM and Random animations.

	Main effect of relationship			Main effect of animation			Interaction		
	*F*	*p*	$\eta_{p}^{2}$	*F*	*p*	$\eta_{p}^{2}$	*F*	*p*	$\eta_{p}^{2}$
Biological	1.998	0.172	0.09	1.001	0.329	0.05	2.545	0.126	0.11
Emotional	0.568	0.459	0.03	0.991	0.331	0.05	0.137	0.715	0.01
Cognitive	1.144	0.297	0.05	1.062	0.315	0.05	2.229	0.150	0.10

### Measures of agency attributions to ball and doll

Table 5 showed average score and standard deviation of children with and without ICs in their agency attributions to ball and doll. We conducted group (IC/NIC) × item (ball/doll) ANOVAs, respectively, on scores for each property (biological, psychological and perceptual) and used PPTV scores as a covariate. For biological properties, the interaction between group and item was significant. Further analysis of simple effects revealed that children with ICs attributed marginally more biological properties to the ball than children without ICs [*F* (1,101) = 3.74, *p* = 0.056, $\eta_{p}^{2}=0.04$ ]. However, children with and without ICs did not differ in their agency attributions to the doll [*F* (1,101) = 0.25, *p* > 0.50, $\eta_{p}^{2}=0.00$ ]. For psychological properties, children with ICs attributed more psychological properties to the ball and the doll compared with children without ICs. More details could be seen in Table 6.

**Table 5:** Mean and standard deviation of agency attributions to ball and doll for imaginary companion status.

	With ICs (*N* = 23)		Without ICs (*N* = 81)	
	*M*	SD	*M*	SD
Ball-biological	1.22	1.126	0.68	0.960
Ball-psychological	1.61	1.076	0.98	1.072
Ball-perceptual	1.04	1.186	0.62	1.067
Doll-biological	1.13	1.180	1.25	1.290
Doll-psychological	1.70	1.185	1.25	1.031
Doll-perceptual	1.39	1.305	1.52	1.266

**Table 6:** Comparisons between children with and without ICs in agency attributions to ball and doll.

	Main effect of group			Main effect of item			Interaction		
	*F*	*p*	$\eta_{p}^{2}$	*F*	*p*	$\eta_{p}^{2}$	*F*	*p*	$\eta_{p}^{2}$
Biological	0.398	0.530	0.00	0.571	0.452	0.01	6.549*	0.012	0.06
Psychological	4.732*	0.032	0.05	0.382	0.538	0.00	0.508	0.477	0.01
Perceptual	0.286	0.594	0.00	3.494	0.064	0.03	3.006	0.086	0.03

* *p* < 0.05: Annotation.

Table 7 showed average score and standard deviation of children with egalitarian and hierarchical IC relationships in their agency attributions to ball and doll. Relationship (egalitarian/hierarchical) × item (ball/doll) ANOVAs were conducted on each property (biological, psychological and perceptual), respectively. For each property, the main effects of item, relationship and the interaction between relationship and item were not significant. More details could be seen in Table 8.

**Table 7:** Mean and standard deviation of agency attributions to ball and doll for child-IC relationship quality.

	Egalitarian relationship (*N* = 12)		Hierarchical relationship (*N* = 11)	
	*M*	SD	*M*	SD
Ball-biological	1.50	1.000	0.91	1.221
Ball-psychological	1.75	1.215	1.45	0.934
Ball-perceptual	1.50	1.168	0.55	1.036
Doll-biological	1.25	1.138	1.00	1.265
Doll-psychological	1.83	1.030	1.55	1.368
Doll-perceptual	1.67	1.231	1.09	1.375

**Table 8:** Comparisons between children with hierarchical and egalitarian IC relationships in agency attributions to ball and doll.

	Main effect of relationship			Main effect of item			Interaction		
	*F*	*p*	$\eta_{p}^{2}$	*F*	*p*	$\eta_{p}^{2}$	*F*	*p*	$\eta_{p}^{2}$
Biological	0.896	0.355	0.04	0.178	0.678	0.01	0.816	0.377	0.04
Psychological	0.451	0.509	0.02	0.185	0.672	0.01	0.000	0.985	0.00
Perceptual	2.816	0.108	0.12	2.729	0.113	0.12	0.772	0.389	0.04

## Discussion

### Comparisons between children with and without ICs in agency attributions

Our first goal was to explore the potential differences between children with and without ICs in their agency attributions to non-human items under a Chinese cultural background. Based on previous researchers’ theory that children with ICs have a strong tendency to anthropomorphize than children without ICs (Tahiroglu, 2012), we predicted that children with ICs might attribute more biological, emotional and cognitive properties to non-human items with behavioral cues, and more biological, psychological and perceptual properties to non-human items with appearance characteristics than children without ICs.

In terms of behavioral cues, we found that children with ICs attributed more cognitive properties to Random and ToM animations than children without ICs. This result is inconsistent with some previous researches. Moriguchi et al. (2016b) found that children with ICs may be more likely than children without ICs to attribute more biological properties to Random animations. Meyer and Tuber (1989) found that children with ICs had much more human, animal and inanimate movement responses than children without ICs. This result indicated that children with ICs may be more sensitive to the movement (one of biological properties) of human, animal and inanimate item compared with children without ICs. Researchers should investigate the reason for these inconsistencies in the future.

In terms of appearances characteristics, we found that children with ICs attributed marginally more biological properties to the ball and more psychological properties to the ball and the doll than children without ICs. The difference in psychological properties is consistent with the finding that children with ICs tended to choose a puppet who had anthropomorphic statements of non-human items (e.g., ‘I think flowers can have personalities’) more than a puppet who disagreed with the statements compared with children without ICs (Tahiroglu, 2012). However, Moriguchi and Shinohara (2012) found that children with and without ICs did not differ in their attributions of biological, psychological and perceptual properties to human and ball. The reason for this inconsistency needs further researches.

In general, our results suggested that children with ICs may be more likely to attribute some agency properties to non-human items with behavioral cues or appearance characteristics than children without ICs. The first hypothesis was partly supported. As mentioned in the Introduction section, previous researchers proposed two explanations from the perspective of ICs. One possible explanation is that children with ICs have a strong tendency to anthropomorphize compared with children without ICs (Tahiroglu, 2012). As a consequent, children with ICs may extend this anthropomorphic tendency to some non-human items with behavioral cues or appearance characteristics. The other possible explanation is that children with ICs have a more sensitive agent perception system than children without ICs (Moriguchi and Shinohara, 2012). We speculated that the creations of ICs and the interactions with ICs may give children more chances to practice their agent perception systems. Thus, children with ICs may be more sensitive to behavior cues or appearance characteristics of some non-human items than children without ICs. In addition, the ability to attribute mental states to other agents was impaired among people with autism (Zwickel et al., 2011). Based on our results, we speculated that pretend play may be associated with some remissions of the impairments among people with autism in agency attributions.

Furthermore, we had proposed in the Introduction section that having an IC, the tendency to anthropomorphize and sensitivity of the agent perception system may share the same core content-dual representation. From one side, since IC play is an instance of pretend play (Leslie, 1987), it may involve a lot of distinctions between reality and fantasy and children with ICs may have a good ability of dual representation. From the other side, when children have a stronger tendency to anthropomorphize or a more sensitive agent perception system, they may attribute more agencies (fantasy) to non-human items (reality), which also means they may have a good ability of dual representation. Thus, we speculate that children who have ICs may be more likely to have a stronger tendency to anthropomorphize or a more sensitive agent perception system compared with other children.

However, our results revealed that the differences between children with and without ICs in their agency attributions might emerge more in the lower level (the ball) than in the higher level (the doll) of appearance characteristics. As to this, we speculated that the doll had more agency cues (e.g., face, clothes) than the ball. Thus, when facing a doll, it was easier for children without ICs to anthropomorphize or detect agencies. This might reduce the differences between children with and without ICs in their agency attributions to non-human items with higher level of appearance characteristics.

### Comparisons between children with hierarchical and egalitarian IC relationships in agency attributions

Our second goal was to explore the potential difference between children with egalitarian and hierarchical IC relationships in their agency attributions to non-human items. Previous researchers proposed that egalitarian IC relationships may have a higher demand for knowing mental states of ICs than hierarchical IC relationships (Lin et al., 2020b). Based on this idea, we speculated that children with egalitarian IC relationships may have a stronger tendency to anthropomorphize than children with hierarchical IC relationships. Children with egalitarian IC relationships may extend this anthropomorphic tendency to other agents. Thus, we predicted that children with egalitarian IC relationships might attribute more biological, emotional and cognitive properties to non-human items with behavioral cues, and more biological, psychological and perceptual properties to non-human items with appearance characteristics compared with children with hierarchical IC relationships. To our surprise, we found that children with egalitarian and hierarchical IC relationships did not differ in their agency attributions to non-human items with behavioral cues or appearance characteristics. Our second hypothesis was not supported by these results.

As to these insignificant results, we had two possible explanations. One possible explanation is that children with egalitarian and hierarchical IC relationships have almost the same chance to anthropomorphize their ICs and they may further extend this anthropomorphic trend to other non-human items. From one side, in the egalitarian relationships, children and their ICs are equal in status (Lin et al., 2018b). Previous researchers proposed that imaginary relationships (e.g., ICs, diary friends) are often associated with emotions (Gleason, 2013) and ICs often have distinct personalities (Rucińska, 2022). Thus, we further speculated that children need to know the physiological and psychological states of ICs, in order to better maintain the egalitarian IC relationships. From the other side, in the hierarchical relationships, children tend to dominate, while ICs are in a relatively weaker status (Lin et al., 2018b). Children need to attribute some properties (e.g., biological, psychological) to the ICs, aiming to let the ICs better serve them. In conclusion, children with different relationship qualities may all need to attribute some agencies to their ICs during the interaction with ICs. The other possible explanation is that children with egalitarian and hierarchical IC relationships may not differ in the sensibility of their agent perception systems. In other words, children with different relationship qualities may have the same chances to practice their agent perception systems.

In general, our results suggest that having an IC of any kind is related to agency attributions to non-human items with behavioral cues and appearance characteristics rather than child-IC relationship qualities, which is inconsistent with the notions that different child-IC relationship qualities may result in some differences in children’s social developments and children with egalitarian IC relationship may have some advantages in social developments than children with hierarchical IC relationships (Gleason, 2004; Gleason and White, 2005). Meanwhile, in the Introduction section, based on previous researchers’ opinion that egalitarian IC relationships may have a higher demand for knowing mental states of ICs than hierarchical IC relationships (Lin et al., 2020b), we speculated that children with egalitarian IC relationships may have a stronger tendency to anthropomorphize than children with hierarchical IC relationships. This speculation seems to be not applicable to our results relating to child-IC relationship qualities. In light of the results in this study, we further speculated that having an IC of any kind may be more related to some dimensions of children’s social developments than child-IC relationship qualities. In regardless of the details in the interactions between children and their ICs, ICs may have some positive influences on some dimensions of children’s social developments as a whole.

### Limitations and directions for future research

We acknowledge three limitations in our study: firstly, although we found that Chinese children’s IC status was associated with their agency attributions to non-human items, this study could not determine the causal directions between them. Specifically, this study could not determine whether the interaction with ICs resulted in attributing more agencies to non-human items, or attributing more agencies to non-human items leaded to the creations of ICs. Secondly, given that researcher only interviewed part of mothers, we might have missed several children who initially did not say they had ICs but who would have acknowledged an IC if asked about one reported by mother (see Taylor and Carlson, 1997). In addition, the definition about ICs which had been provided for mothers may not include the instances that the child only talked to others about the IC, but never play with it. This definition might have changed mothers’ answers. Meanwhile, since we only classified 23 children as having ICs, the sample size of children with egalitarian (*N* = 12) and hierarchical (*N* = 11) IC relationships was relatively small. As mentioned above, the power (1-β) for the relationship was relatively low. Our thus, the sample size might not be large enough for us to explore the potential differences between children with egalitarian and hierarchical IC relationships in their agency attributions. Thirdly, it was the first time that we know of to use the Frith-Happé animations (Castelli et al., 2000, 2002) among Chinese children. Thus, the animation stimuli or the presenting mode might be not quite appropriate for Chinese children which would influence our results. Meanwhile, given that researcher introduced the ball or the doll as his or her good friend, children might simply assume that the researcher was pretending and go along with the pretense, which might influence children’s agency attributions. However, we speculated that this influence might be same to children with and without ICs, and children with hierarchical and egalitarian IC relationships.

Here, we think that there are three directions for further researchers. The first, in order to explore the potential differences of agency attributions between children with and without ICs, and between children with hierarchical and egalitarian IC relationships in a more accurate way, future researchers should further enlarge the sample size. The second, although one previous study investigated the influences of children’s agency attributions on their IC status (Moriguchi et al., 2016a), future researchers can still use a cross lag study to investigate the causal directions between children’s IC status and their agency attributions. The third, if one of the causal directions or both the causal directions exist, it is interesting for future researchers to explore the underlying mechanism. For example, previous research found that children with ICs have a better emotion understanding and theory of mind compared with children without ICs (Giménez-Dasí et al., 2014). We speculate that ICs may have some positive effects on children’s social cognition abilities (e.g., theory of mind and emotion understanding) and then children with higher social cognition abilities may be more sensitive to some agency cues. Finally, children with ICs may attribute more agencies to non-human items with these cues than children without ICs. Thus, social cognition ability may play the mediating role between children’s IC status and their agency attributions.

## Conclusion

The value of this study is two folds. The first, these findings deepen our understanding of the relationship between children’s IC and their goal-directedness of agencies. The second, these findings give some explanations to the previous findings that children with ICs had better peer relationships than children without ICs (Lin et al., 2018a). As to imaginary relationships, previous researchers hold two different opinions. One point is that imaginary relationships include an imagined sense of reciprocity and are often associated with emotions (Gleason, 2013). The other point is that enactivism proposes thinking of imaginary friend play as an explicitly embodied and performative act, where one does not need to have mental representations about absent entities (Rucińska, 2022). However, this inconsistency needs further discussions in the future. Since children with ICs may be more likely to attribute some agencies to non-human items than children without ICs, we speculate that children with ICs may have more interactions with non-human items other than imaginary partners. In other words, compared with children without ICs, children with ICs may have more mental rehearsals of thinking about these non-human items’ minds, which may result in better peer relationships.

## Data availability statement

The original contributions presented in the study are included in the article/Supplementary material, further inquiries can be directed to the corresponding author.

## Ethics statement

The studies involving human participants were reviewed and approved by Academic Ethics Committee of Huaiyin Normal University. Written informed consent to participate in this study was provided by the participants’ legal guardian/next of kin.

## Author contributions

LQ participated in the design, data collection, data analysis, data interpretation, and drafting the early and final version of the article. ZR participated in the design, data collection, data analysis, data interpretation, and drafting the early version of the article. ZY participated in data collection and wrote the computer program that was used to present random animations or ToM animations. ZN participated in the design, data analysis, data interpretation, and gave some advises on the early and final version of the article. All authors contributed to the article and approved the submitted version.

## Funding

This work was supported by the Jiangsu Province Education Science Planning Project [C-b/2021/01/40]. This fund will pay for the open access publication fees.

## Conflict of interest

The authors declare that the research was conducted in the absence of any commercial or financial relationships that could be construed as a potential conflict of interest.

## Publisher’s note

All claims expressed in this article are solely those of the authors and do not necessarily represent those of their affiliated organizations, or those of the publisher, the editors and the reviewers. Any product that may be evaluated in this article, or claim that may be made by its manufacturer, is not guaranteed or endorsed by the publisher.

## Footnotes

^1^We also removed these 16 children into the children with ICs group and made some further analyses. The results revealed that children with ICs scored higher on the PPVT compared with children without ICs, (*t* (102) = −2.11, *p* < 0.05, *Cohens’ d* = −0.43). In terms of appearance characteristics, children with ICs attributed more psychological properties to the ball and the doll than children without ICs [*F* (1,101) = 6.21, *p* < 0.05, *η*_p_^2^ = 0.06]. The main effect for item (ball/doll) was significant for perceptual properties [*F* (1,101) = 6.76, *p* < 0.05, *η*_p_^2^ = 0.06]. These results were a little different from the following results that included the 16 children in the children without ICs group.

## Supplementary material

SUPPLEMENTARY FIGURE 1 Picture one used in the imaginary companion interview for children (one story about an invisible friend).

SUPPLEMENTARY FIGURE 2 Picture two used in the imaginary companion interview for children (one story about a personified object).

SUPPLEMENTARY FIGURE 3 Picture three used in the imaginary companion interview for children (one story about a personified object).
