# Supplementary figures and images for: Did Chinese children with imaginary companions attribute more agencies to non-human items: Evidences from behavioral cues and appearance characteristics
Source: Front Psychol. 2022 Sep 7;13:899047. doi: 10.3389/fpsyg.2022.899047 (PMC9491396; doi:10.3389/fpsyg.2022.899047)

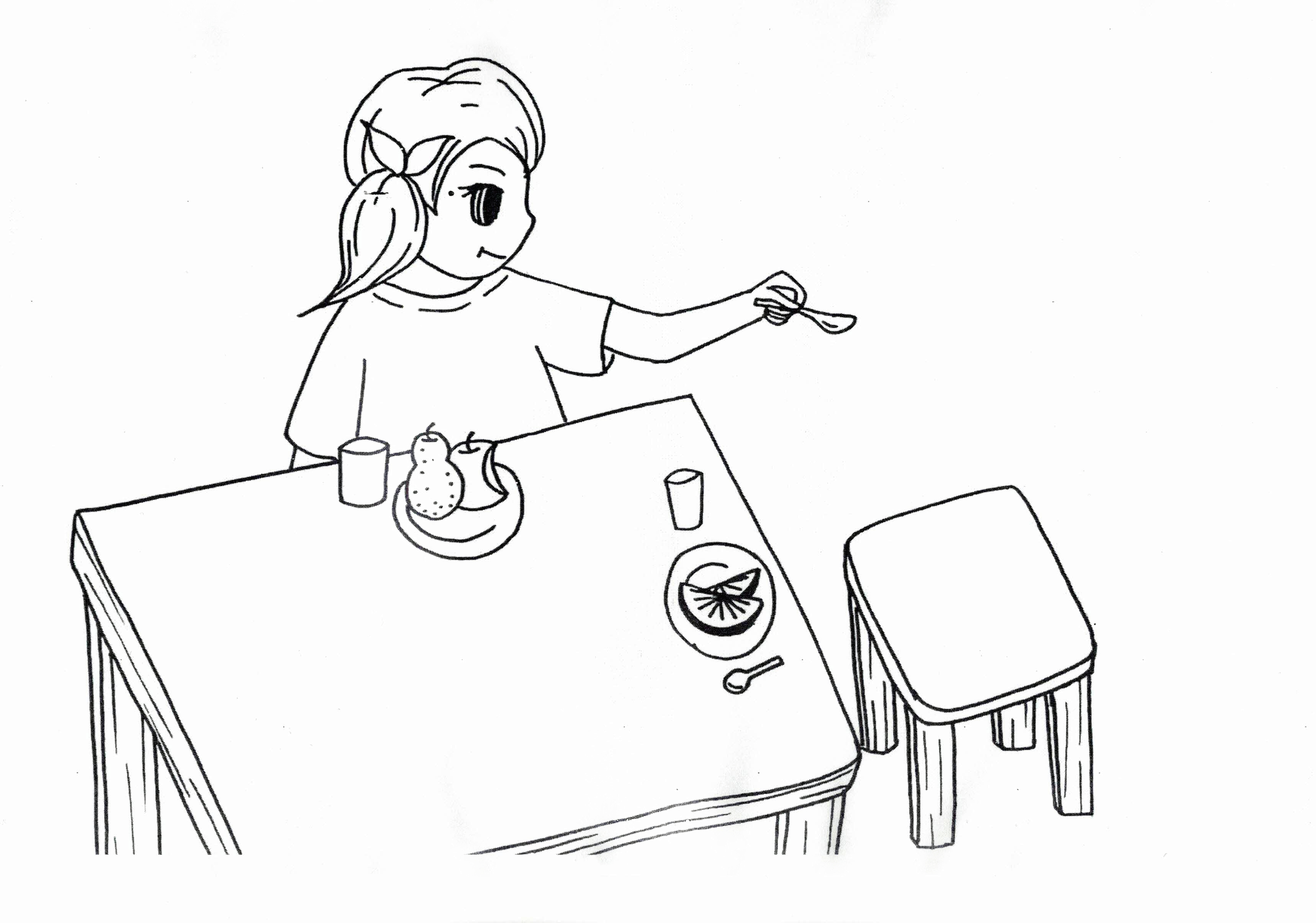

Supplement: SUPPLEMENTARY FIGURE 1 — Picture one used in the imaginary companion interview for children (one story about an invisible friend). [file Image_1.JPEG]

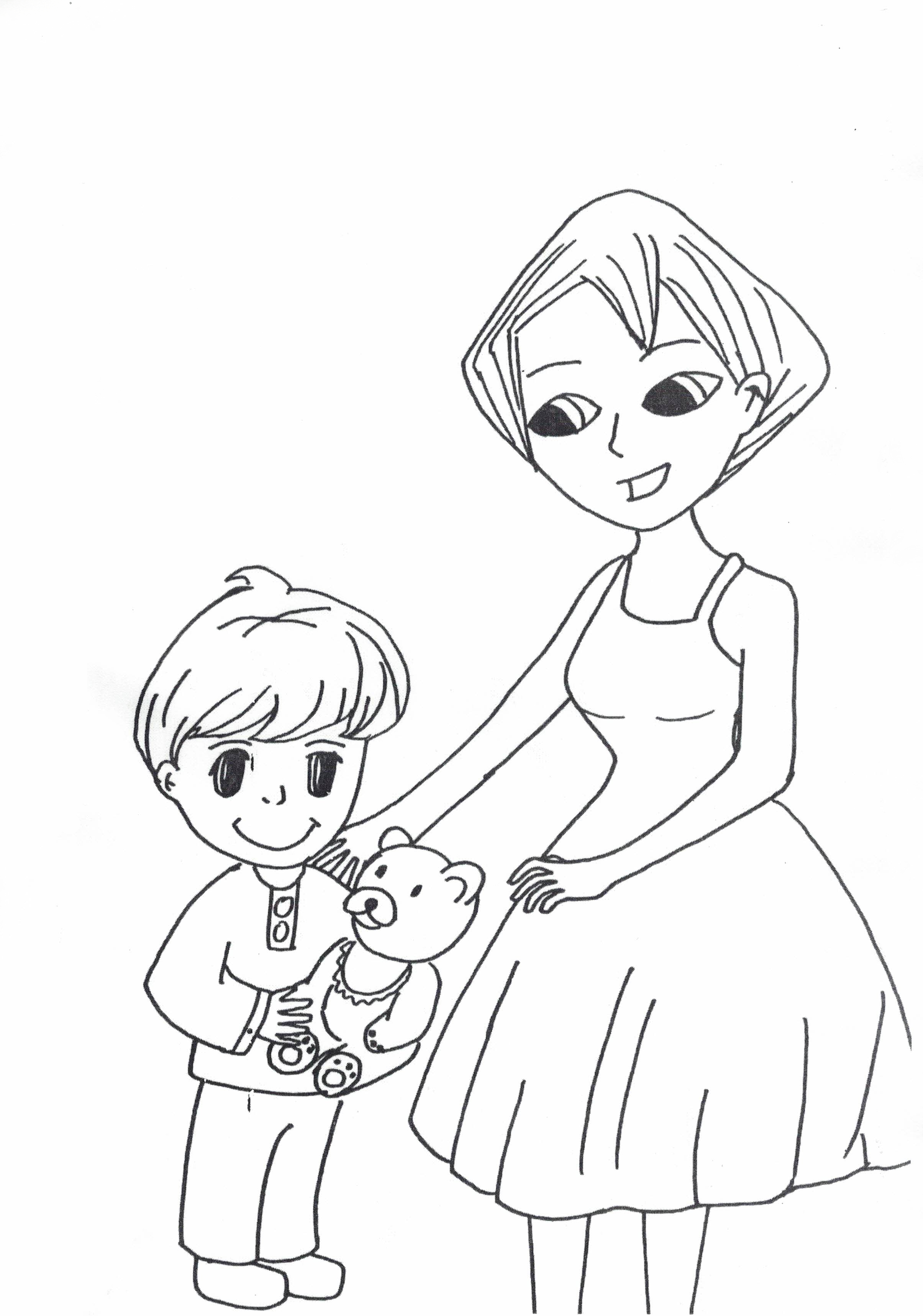

Supplement: SUPPLEMENTARY FIGURE 2 — Picture two used in the imaginary companion interview for children (one story about a personified object). [file Image_2.JPEG]

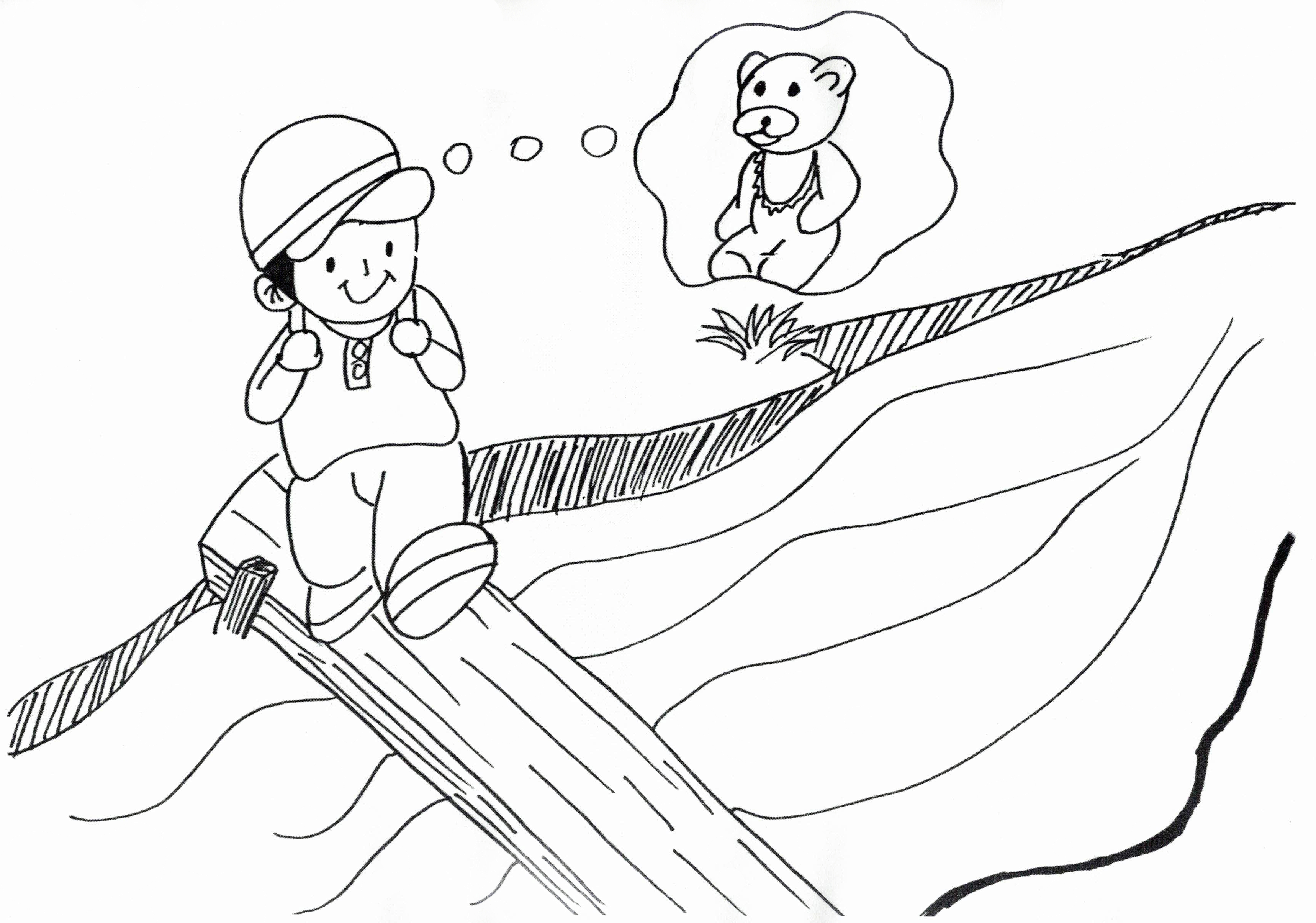

Supplement: SUPPLEMENTARY FIGURE 3 — Picture three used in the imaginary companion interview for children (one story about a personified object). [file Image_3.JPEG]
